# Pathotyping the Zoonotic Pathogen Streptococcus suis: Novel Genetic Markers To Differentiate Invasive Disease-Associated Isolates from Non-Disease-Associated Isolates from England and Wales

**DOI:** 10.1128/JCM.01712-18

**Published:** 2019-06-25

**Authors:** Thomas M. Wileman, Lucy A. Weinert, Kate J. Howell, Jinhong Wang, Sarah E. Peters, Susanna M. Williamson, Jerry M. Wells, Paul R. Langford, Andrew N. Rycroft, Brendan W. Wren, Duncan J. Maskell, Alexander W. Tucker

**Affiliations:** a Department of Veterinary Medicine, University of Cambridge, Cambridge, United Kingdom; b Animal and Plant Health Agency, Bury St Edmunds, United Kingdom; c Host-Microbe Interactomics, Department of Animal Sciences, Wageningen Univeristy, Wageningen, the Netherlands; d Section of Paediatrics, Department of Medicine, Imperial College London, London, United Kingdom; e The Royal Veterinary College, Hawkshead Campus, Hatfield, United Kingdom; f Faculty of Infectious & Tropical Diseases, London School of Hygiene & Tropical Medicine, London, United Kingdom; University of Tennessee at Knoxville

**Keywords:** molecular diagnostics, pathotyping, *Streptococcus suis*, surveillance, virulence markers

## Abstract

Streptococcus suis is one of the most important zoonotic bacterial pathogens of pigs, causing significant economic losses to the global swine industry. S. suis is also a very successful colonizer of mucosal surfaces, and commensal strains can be found in almost all pig populations worldwide, making detection of the S. suis species in asymptomatic carrier herds of little practical value in predicting the likelihood of future clinical relevance. The value of future molecular tools for surveillance and preventative health management lies in the detection of strains that genetically have increased potential to cause disease in presently healthy animals. Here we describe the use of genome-wide association studies to identify genetic markers associated with the observed clinical phenotypes (i) invasive disease and (ii) asymptomatic carriage on the palatine tonsils of pigs on UK farms. Subsequently, we designed a multiplex PCR to target three genetic markers that differentiated 115 S. suis isolates into disease-associated and non-disease-associated groups, that performed with a sensitivity of 0.91, a specificity of 0.79, a negative predictive value of 0.91, and a positive predictive value of 0.79 in comparison to observed clinical phenotypes. We describe evaluation of our pathotyping tool, using an out-of-sample collection of 50 previously uncharacterized S. suis isolates, in comparison to existing methods used to characterize and subtype S. suis isolates. In doing so, we show our pathotyping approach to be a competitive method to characterize S. suis isolates recovered from pigs on UK farms and one that can easily be updated to incorporate global strain collections.

## Abstract (precis)

Streptococcus suis is one of the most important zoonotic bacterial pathogens of pigs, causing significant economic losses to the global swine industry. S. suis is also a very successful colonizer of mucosal surfaces, and commensal strains can be found in almost all pig populations worldwide, making detection of the S. suis species in asymptomatic carrier herds of little practical value in predicting the likelihood of future clinical relevance.

## INTRODUCTION

Streptococcus suis is one of the most important bacterial pathogens of pigs, causing significant economic losses to the swine industry worldwide (1). The infectious agent is responsible for a wide range of clinical manifestations, including septicemia with sudden death, meningitis, endocarditis, arthritis, and pneumonia, among other diseases (2). S. suis is also a zoonotic pathogen associated with exposure to pigs or pork-derived products (3). Although cases in Europe are infrequently reported, in recent years the surveillance and number of reported human infections have increased substantially in Southeast Asia (4–9).

Importantly, S. suis is not only an invasive pathogen but also a very successful colonizer of mucosal surfaces (10). In fact, the upper respiratory tract of pigs, in particular the palatine tonsils, is considered to be both the natural habitat of S. suis and a principal route of invasion, although the bacterium can also be recovered from the gastrointestinal and genital tracts (2). Colonization of adult pigs is common in almost all pig populations sampled, meaning that transfer of S. suis from sow to piglet during parturition and suckling is an important route of transmission (10).

Several methods exist to investigate strain diversity and identify phylogenetic groups of S. suis. Simple biochemical tests cannot always differentiate S. suis from S. suis-like strains when performed on cultured isolates recovered from diseased animals and to date remain of little practical use in differentiating invasive disease-associated strains from asymptomatic commensal-like strains, both of which may contribute subclinically to the respiratory microflora of colonized pigs (1). Other existing methods used to characterize and subtype S. suis as part of epidemiological studies have recently been the subject of a comprehensive review by Xia et al. (11). Each approach has its limitations, often requiring either large amounts of sample DNA, which are labor-intensive and cumbersome to obtain, or high levels of technical competence, making the comparison of results between laboratories difficult.

To date, serotyping remains the most widely used method to subtype S. suis isolates and is an important part of the routine diagnostic procedure (2, 12). A total of 35 serotypes have been described for S. suis based on differences in the capsular polysaccharide antigens, but since their original descriptions, evidence has emerged for the reclassification of a number of serotypes as other *Streptococcus* species, meaning that current opinion considers there to be just 29 “true” S. suis serotypes (namely, 1 to 19, 21, 23 to 25, 27 to 31, and 1/2) (13). Serotype 2 predominates among clinical cases of disease in most countries, although serovars 1 to 9, 14, and 1/2 have all been documented as being of clinical importance in certain geographical locales (14–18). As a result, serotyping has been used as a proxy for predicting the virulence potential of S. suis isolates. However, the use of serotyping alone as a predictor of virulence has the limitation that strains of the same serotype can vary substantially in virulence (19, 20).

Given the limitations of serotyping to reliably predict virulence potential of S. suis strains, other markers have been investigated. A wide range of homologs of bacterial virulence factors and virulence-associated factors found in other Gram-positive organisms has been shown to affect the virulence of S. suis strains through targeted-mutagenesis studies (21–23). However, a clear association with specific roles in the onset and development of disease has not been found for many proposed factors (24, 25). Despite this, the “virulence-associated markers” (rather than virulence factors *per se*) extracellular protein factor (EF; encoded by the *epf* gene) (26) and muramidase-released protein (MRP; encoded by the *mrp* gene) (27), as well as the thiol-activated toxin hemolysin suilysin (SLY; encoded by the *sly* gene) (28, 29), have been extensively used to predict the virulence potential of S. suis strains in certain mainly European countries, particularly for strains of serotype 2 (17, 24, 30). Unfortunately, genotyping of *epf, mrp*, and/or *sly* also fails to provide clear classification of a S. suis isolate as virulent (or not) because isogenic mutants devoid of such factors have been found to be as virulent as their respective parental strains, emphasizing the importance of their consideration as virulence-associated markers rather than true virulence factors *per se* (31).

Advances in sequencing technologies now allow whole-genome sequencing (WGS) of multiple strains of the same species, including S. suis (32–36). This explosion in the amount of detailed genetic information has allowed Bayesian analysis of population structure and the investigation of S. suis recombination rates, revealing enormous species diversity and significant genomic differences between S. suis isolates responsible for systemic disease in pigs compared to nonclinical isolates recovered from the upper respiratory tract (35). Indeed, in 2015 Weinert et al. proposed that loss of protein-encoding sequences had led to a smaller systemic disease-associated genome with increased virulence potential and an overrepresentation of genes encoding previously reported virulence factors associated with S. suis (35).

Minimum core genome (MCG) sequence typing is a recently described typing scheme that also takes advantage of the increase in available S. suis WGS data, using population genetics-based subdivisions for strain identification and typing (33, 37). MCG sequence typing exploits advances in next-generation sequencing to identify novel regions of the core genome that can be used to identify and type S. suis isolates into “MCG groups” that can later be associated with clinical phenotypes. In fact, during the design of this method, MCG group 1 was reported as being assigned to all highly virulent isolates tested and associated with the greatest occurrence of previously reported virulence genes (33). However, MCG sequence typing, like multilocus sequence typing (MLST) also described for S. suis (38), is difficult to apply to routine diagnostic testing and can sometimes lack the discriminatory power to differentiate bacterial strains into virulent and avirulent subpopulations, limiting its usefulness in epidemiological studies.

The aim of this study was to design and then evaluate a pathotyping tool to predict the virulence potential of S. suis isolates using genome-wide association studies, a so-far-unexploited method for the identification of S. suis virulence-associated markers. The statistical power to allow the identification of robust associations between genotype and phenotypes, including virulence in many different bacterial species, is now possible due to the rapid increases in the availability of detailed WGS data (39, 40). In this study, we have combined WGS data with high-quality clinical metadata in order to identify genetic markers in the S. suis accessory genome (i.e., genes absent from one or more isolates or unique to a given isolate) associated with (i) invasive disease or (ii) asymptomatic carriage on the palatine tonsils of pigs on UK farms. Subsequently, we designed a multiplex PCR (mPCR) to target three genetic markers that differentiated 115 S. suis isolates into (i) invasive disease-associated and (ii) non-disease-associated groups. We also describe evaluation of our pathotyping tool (generalized linear model and mPCR), using an out-of-sample collection of 50 previously uncharacterized S. suis isolates, in comparison to existing methods used to characterize and subtype S. suis isolates. In doing so, we show our approach to be a competitive method to subtype S. suis isolates recovered from pigs on UK farms and one that can easily be updated to incorporate global strain collections.

## MATERIALS AND METHODS

### Bacterial isolates.

Two groups of S. suis isolates were used in this study, (i) a training collection of 115 isolates and (ii) an out-of-sample test collection of 50 previously uncharacterized isolates. The original training collection was used to identify genetic markers which could differentiate S. suis isolates into (i) invasive disease-associated and (ii) non-disease-associated phenotypic groups. The training collection consisted of laboratory reference strain P1/7 (GenBank accession number NC_012925), originally recovered from an antemortem blood culture from a pig that died with meningitis in the United Kingdom (32, 41). The other 114 isolates of the training collection were a subset recovered from pigs on farms in England and Wales during routine diagnostic investigations at the Animal Health and Veterinary Laboratories Agency (AHVLA; now the Animal and Plant Health Agency [APHA]) in 2010, and contribute to a larger collection previously described in 2015 by Weinert et al. (35). Well-defined phenotypic metadata were available based on which each isolate was categorized as being associated with invasive S. suis disease (*n* = 53; recovered from systemic sites in the presence of clinical signs [arthritis, meningitis, and septicemia] and/or gross pathology consistent with S. suis infection) or as being non-disease associated (*n* = 62; recovered from the tonsil or trachea-bronchus of pigs without any typical signs of streptococcal disease but diagnosed with disease unrelated to S. suis, such as enteric disease). The out-of-sample test collection was used to evaluate our pathotyping tool. Out-of-sample forecasting is a common approach used to evaluate the performance of binary diagnostic tests. To avoid reducing statistical power, rather than split the training collection, an additional out-of-sample “test” collection was put together consisting of 23 invasive disease-associated (recovered from systemic, nonrespiratory locations of pigs diagnosed with S. suis disease at the APHA during 2013) and 27 non-disease-associated isolates (recovered from material scraped from the palatine tonsils of pigs exhibiting no signs of S. suis disease on farms in England and Wales between June 2013 and May 2014). Sites of recovery and ante- and postmortem findings of all isolates described in this study are summarized in Table S1 in the supplemental material.

### Identification of genetic markers associated with observed clinical phenotype.

Genetic markers to pathotype S. suis were identified using positive detection data of putative protein-encoding sequences making up the S. suis accessory genome (i.e., genes absent from one or more isolates or unique to a given isolate). The accessory genome was taken from Weinert et al. (35). Briefly, *de novo* assemblies of Illumina fastq reads were produced and protein-encoding genes identified, that were then used in Markov clustering to find orthologue groups. Two complementary genome-wide association studies, (i) the univariate chi-squared test for independence and (ii) the multivariate discriminant analysis of principal components (DAPC), were combined to define a preliminary list of genetic markers associated with the observed clinical phenotypes (i) invasive disease and (ii) asymptomatic carriage on the palatine tonsils of pigs. The chi-squared test for independence, implemented in the R package *stats* (42), was used to compare the observed positive detection of protein-encoding sequences with expected frequencies, in doing so calculating a test statistic that if greater than the critical value was reason to reject the null hypothesis of independence (*P* value < 0.05). Bonferroni adjustment (α/*n*) was used to control for family-wise error associated with multiple sampling.

DAPC (43, 44), implemented in the R package *adegenet* (45, 46), was used to identify genetic differences between predefined phenotypic groups. The total amount of original variation retained in the DAPC model affected which genetic markers contributed most to the separation of genetic structures. As a result, four independent DAPC analyses were performed retaining 60, 70, 80, or 90% of the original genetic variation, and the 1% of ranked genetic markers contributing most to the discrimination of predefined phenotypic groups was then analyzed and genetic markers consistently output by two or more DAPC analyses taken forward as candidates for pathotyping S. suis.

### Analysis of the distribution of previously reported virulence factors associated with S. suis disease.

Protein-encoding sequences present in P1/7, taken from the list of previously published virulence and virulence-associated factors compiled as part of a comprehensive review by Fittipaldi et al. (24), were extracted from GenBank (Table S2). P1/7 protein-encoding sequences were used as tBLASTn queries against a bespoke BLAST database consisting of the draft genome assemblies of all isolates described in this work. Amino acid level matches to >80% of >80% of the total length of each translated protein-encoding sequence were considered hits.

### Selection of genetic markers to pathotype S. suis.

Logistic regression analysis in the form of a generalized linear model (GLM) with backwards-stepwise selection using penalized likelihood ratio tests, implemented in the R package *logistf* (47), was used to identify the fewest statistically significant (*P* value < 0.05) markers to differentiate S. suis isolates into predefined (i) invasive disease-associated and (ii) non-disease-associated groups. A receiver operating characteristic (ROC) curve, implemented in the R package *ROCR* (48), was used to visualize the GLM performance metrics true-positive rate (sensitivity) and false-positive rate (1 − specificity) in comparison to the observed clinical phenotype (considered to be the “gold standard” in this study), and a cutoff threshold was selected to convert the real-valued output (fitted values) of the logistic regression (probability of causing invasive disease) into a binary class decision: invasive disease-associated (1)/non-disease associated (0). As no cutoff was optimal according to all possible performance criteria, the cutoff choice involved a trade-off between different performance metrics where a low false-negative rate (1 − sensitivity, analogous to type II error) was chosen as the most valuable performance metric for pathotyping S. suis, with a view to establish and then maintain a pig population free of invasive disease-associated strains.

All statistical analyses were performed using the standard R environment for statistical computing and graphs (version 3.1.1) (49).

### Identification of S. suis-species specific genetic markers.

We designed an mPCR to target genetic markers associated with observed clinical phenotype, along with an S. suis species-specific marker as a positive control. The most conserved protein-encoding sequences of the S. suis core genome (i.e., present in all isolates) were used to select a species-specific marker to complement the pathotyping markers. To do this, all annotated protein-encoding sequences of S. suis strain P1/7 were used as BLASTn queries against a bespoke BLAST database of all *de novo* assemblies and known S. suis complete genome sequences. Protein-encoding sequences with identities of >95% across >80% of the total length of each query sequence were then used to query the NCBI nonredundant (nr) database to identify matches only to S. suis.

### Multiplex PCR and detection of PCR amplicons.

The software primer3 version 4.0.0 (http://primer3.ut.ee) was used to design mPCR primers. All mPCR primers were designed to target conserved regions within the protein-encoding sequence of genetic markers (as opposed to flanking regions) and are summarized in Table 1. Primers were designed to have similar physical characteristics, enabling simultaneous amplification under the same thermal cycling conditions and in multiplex reactions. Primer length (21 to 30 bp), GC content (40 to 60%), melting temperature (>68°C if possible but at least 60°C), and expected amplicon size (100 to 1,000 bp) were based on the manufacturer’s recommendations for primer design using the Multiplex PCR *Plus* kit (Qiagen). Consistency between the positive detection of genetic markers and primer matches was investigated using BLASTn. Prior to ordering, all primers were queried against the NCBI nr nucleotide database to check for non-S. suis DNA matches. Primers were synthesized by Sigma-Aldrich (Haverhill, United Kingdom) and delivered in solution (TE buffer; 10 mM Tris-Cl, 1 mM EDTA [pH 8.0]) at a stock concentration of 100 μM; primers were used at a working stock concentration of 20 μM.

**TABLE 1:** Multiplex PCR primer details^*a*^

Primer name	Primer sequence (5′–3′)	Marker of:	Multiplex PCR amplicon size (bp)	Predicted biological function (Interpro)
SSU0207_0735F	TTACAAGAACAGGGCAAGACAGTCGCC	Disease association	211	Copper exporting ATPase 1
SSU0207_0945R	GCTGCTTTATAATCTGGGTGTTCGTTG			
SSU1589_0460F	CCTTTAATGCAGGGGACAAAAGTGAGCTC	Disease association	347	Type I restriction-modification system S protein
SSU1589_0806R	CCCATAATCTTACAGTTAACTTCCTTGC			
SSUST30534_0368F	ATCCCCTCCCAATAAAAGATTTGGATGC	Non-disease association	892	Putative sugar ABC transporter
SSUST30534_1259R	TTTTCGAGCTCTCCATACACTGCTTCTG			
SSU0577_0086F	CAGGTAGTTTGGGCTTAGCTTCATCAGG	*Streptococcus suis*	722	Sporulation regulator (WhiA)
SSU0577_0807R	TGGATGCTGAATTCGCAACTGGGCAATC			

a Multiplex PCR primers were designed using the software primer3 (version 4.0.0) and designed to target conserved regions within the protein-encoding sequence of genetic markers (as opposed to flanking regions). Primers were designed to have similar physical characteristics, enabling simultaneous amplification under the same thermal cycling conditions and in multiplex reactions. GenBank identifier prefixes “SSU” and “SSUST3” correspond to Streptococcus suis P1/7 (NC_012925) (32) and Streptococcus suis ST3 (NC_015433) (71), respectively.

All mPCRs were performed using the Multiplex PCR *Plus* kit (Qiagen) and, unless otherwise stated, contained the same reagents except for template DNA. The reaction mixture (50 μl) for each mPCR consisted of 25 μl of 2× Multiplex PCR master mix, 5 μl of 10× CoralLoad dye, 10 μl of RNase-free water, 0.2 μM (final concentration) of each primer, and 10 ng of template DNA. The three-step thermal cycling program for all reactions was as follows: 95°C for 5 min, followed by 35 cycles of 95°C for 30 s (denaturation), 66°C for 90 s (annealing), and 72°C for 90 s (extension), with a final extension of 68°C for 10 min using a T100 thermal cycler (Bio-Rad).

PCR products were analyzed by gel electrophoresis using 2% (wt/vol) UltraPure agarose (Invitrogen) gels made with 1× Tris-borate-EDTA (TBE) buffer and containing 1× SYBR Safe DNA gel stain (Invitrogen). Running time was 60 min at a constant 100 V. Results were visualized using a GelDoc imager (Bio-Rad). Where appropriate, mPCR products were purified using the QIAquick PCR purification kit (Qiagen) as per the manufacturer’s instructions and Sanger sequenced using the Source Bioscience Lifesciences sequencing service. Returned sequencing data were aligned with reference sequences of the target protein-encoding sequence using CodonCode Aligner software (CodonCode Corporation).

The approximate limit of detection of the mPCR was estimated from 10-fold serial dilutions of S. suis genomic DNA of known concentration. DNA extracted from four isolates of the training collection representing invasive disease-associated (SS002 and SS004) and non-disease-associated (LSS011 and LSS027) phenotypes/genotypes was mixed in equal quantities so that templates for each mPCR amplicon would be present in all reactions. A series of 10-fold dilutions was then performed to create mPCR templates of decreasing concentration. The limit of detection was considered to be the lowest concentration of template DNA from which all predicted mPCR amplicons, after 35 thermal cycles, were easily visible under UV transillumination.

To evaluate the specificity of the mPCR assay for S. suis, field isolates of *Streptococcaceae* commonly recovered from the upper respiratory tract of pigs on farms in England and Wales were used as a panel of negative controls. The collection included isolates of Streptococcus gallolyticus, Streptococcus orisratti, Streptococcus pneumoniae, and Streptococcus uberis, sourced from Biotechnology and Biological Sciences Research Council (BBSRC) research project BB/L003902/1. In addition, commensal *Pasteurellaceae*, including Actinobacillus indolicus, Actinobacillus minor, Actinobacillus porcinus, and Haemophilus parasuis (Nagasaki and SW140), were also included, as well as DNA from an *Alcaligenaceae* isolate, Bordetella bronchiseptica RB50 (GenBank accession number NC_002927) (50).

### Comparison of our pathotyping tool to existing methods used to subtype disease-associated isolates of S. suis.

To compare our pathotyping tool (GLM and mPCR) to published methods used to subtype disease-associated isolates of S. suis, the molecular serotype, virulence-associated gene (*epf*, *mrp*, and *sly*) profile, MLST, and MCG sequence type were all determined *in silico*. For comparison of our pathotyping tool against each existing method, the original training collection was used to “train” a model that was then applied to the out-of-sample test collection.

Traditional serotyping (by capillary precipitation) data were unavailable for all S. suis isolates described in this study; therefore, molecular serotyping was performed using an adaptation (for *in silico* use) of the mPCR assays described by Liu et al. (51). Primer sequences were used as BLASTn queries, and nucleotide level matches to >95% of the total length of each primer sequence were considered hits. The distance between hits was compared to reported PCR amplicon sizes. Isolates that could not be assigned to one of the 35 (1 to 34 and 1/2) originally described S. suis serotypes were deemed nonserotypeable (NT). Differentiation of molecular serotypes 1 from 14 and 2 from 1/2 was performed using the method described by Athey et al. (52). All isolates, in particular those deemed to be NT, were confirmed to be S. suis using a combination of (i) biochemical profile (API 20 Strep), (ii) MLST data, and (iii) *recN* sequence homology (53).

Virulence-associated gene profiling was performed using an adaptation (for *in silico* use) of the method described by Silva et al. (54). Again, mPCR and singleplex PCR primer sequences were used as BLASTn queries and nucleotide level matches to >95% to the total length of each primer sequence were considered hits. The distance between hits was compared to reported PCR amplicon sizes. Logistic regression (as described above) using the prevalence of *epf*, *mrp*, and/or *sly* as the GLM explanatory variables was used to classify all isolates as (i) invasive disease associated or (ii) non-disease associated.

MLST was performed using the software MLST version 2.0 (http://cge.cbs.dtu.dk) (55).

MCG sequence typing was performed using an adaptation (for *in silico* use) of the method described by Zheng et al. (37). Multiplex PCR primer sequences were used as BLASTn queries and nucleotide level matches to >95% of the total length of each primer sequence were considered hits. The distance between hits was compared to reported mPCR amplicon sizes. Nucleotide sequences between primer sequence matches were then extracted aligned against the MCG typing reference strain GZ1 (GenBank accession number CP000837), and the 10 single nucleotide polymorphisms (SNPs) of interest were called, allowing isolates to be assigned to one of the seven reported MCG groups for S. suis.

McNemar’s chi-squared test for count data, implemented in the R package *stats* (42), was used to test for statistically significant differences in the sensitivities and specificities of two binary diagnostic tests in a paired study. The weighted generalized score statistic for comparison of predictive values as proposed by Kosinski (56), implemented in the R package *DTComPair* (57), was used to test for significant differences in (negative and positive) predictive values of two binary diagnostic tests.

## RESULTS

### Design of a pathotyping tool for S. suis.

Genetic markers to pathotype S. suis were identified using positive detection data of 7,261 putative protein-encoding sequences making up the S. suis accessory genome (35). To do this, the output of two complementary genome-wide association studies were combined to define a preliminary list of 497 genetic markers associated with the observed clinical phenotypes (i) invasive disease and (ii) asymptomatic carriage on the palatine tonsils of pigs. A multistep process was used to reduce the preliminary list to a number suitable for logistic regression analysis, retaining genetic markers only if (i) positively detected in >50% of invasive disease-associated and <50% of non-disease-associated isolates (and vice versa, i.e., <50% of invasive disease-associated and <50% of non-disease-associated isolates; *n* = 88 remaining), (ii) the protein-encoding sequence length was >500 bp (based on the manufacturer’s recommendations for primer design using the Qiagen Multiplex PCR *Plus* kit; *n* = 44 remaining), and (iii) not predicted to be a mobile genetic element, such as a phage gene, integrase, or transposon (based on Prokka annotations; *n* = 14 remaining). A GLM with backwards-stepwise selection using penalized likelihood ratio tests was then used for the final selection of genetic markers, two associated with invasive disease and one associated with asymptomatic carriage (Table 1). A receiver operating characteristic (ROC) curve was used to visualize the GLM performance metrics true positive rate (sensitivity) and false-positive rate (1 − specificity) and select the cutoff threshold of 0.43 to be used to convert the real-valued output (fitted values) of the GLM into a binary class decision: invasive disease associated or non-disease associated (Table S1). In comparison to the observed clinical metadata, considered the gold standard in this study, our three genetic markers subtyped the 115 S. suis isolates of the training collection with a sensitivity of 0.91, specificity of 0.79, negative predictive value of 0.91, and positive predictive value of 0.79 (Table S3a).

At present, WGS is not readily available for routine surveillance studies in veterinary diagnostics laboratories; therefore, we designed an mPCR to target the three genetic markers selected to pathotype S. suis. In addition to genetic markers selected to differentiate S. suis isolates into (i) invasive disease-associated and (ii) non-disease-associated groups, we also incorporated an S. suis species-specific marker into our mPCR assay. To do this, we first identified the most conserved protein-encoding sequences contributing to the S. suis core genome (i.e., present in all isolates) and selected SSU0577 as a novel S. suis species-specific marker; this marker had a minimum nucleotide sequence identity of 98.15% across the total length of the 918-bp protein-encoding sequence.

### Evaluation of our pathotyping mPCR with the original training collection.

**FIG 1:**
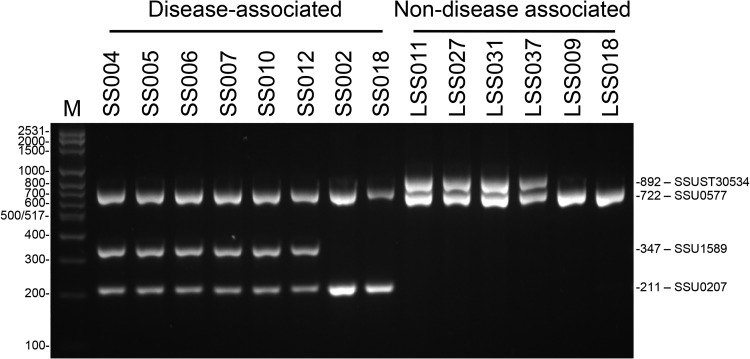
Agarose gel showing the multiplex PCR amplicons produced from genomic DNA of eight invasive disease-associated and six non-disease-associated isolates of S. suis recovered from pigs on farms in England and Wales. PCR amplicons were electrophoresed on a 2% (wt/vol) agarose gel containing 1× SYBR Safe DNA gel stain for 60 min at a constant 100 V and photographed under UV transillumination. Multiplex PCR amplicon patterns matched anticipated amplicon patterns based on *in silico* analyses for all isolates described here. Isolate names are indicated above lanes. Lane M contains 1× Bioline HyperLadder 100 bp Plus DNA ladder, with sizes indicated on the left (base pairs). Multiplex PCR amplicon sizes are indicated on the right (base pairs).

Figure 1 shows an example of the mPCR amplicon patterns after gel electrophoresis on a 2% (wt/vol) agarose gel and photographed under UV transillumination. Amplicons of 722 bp correspond to the S. suis species-specific marker (SSU0577) and were produced by all isolates of the training collection irrespective of invasive disease-associated/non-disease-associated phenotype or genotype. Other amplicons of 211 bp and 347 bp correspond to the invasive disease-associated markers SSU0207 and SSU1589, respectively, and amplicons of 892 bp correspond to the non-disease-associated marker SSUST30534.

To determine the analytical sensitivity of the mPCR, the approximate limit of detection was estimated from 10-fold serial dilutions of S. suis genomic DNA of known concentration. The limit of detection was estimated to be ∼0.0001 ng of S. suis genomic DNA (equivalent to ∼45 genome copies), the lowest concentration of template DNA from which all predicted mPCR amplicons, after 35 thermal cycles, were easily visible under UV transillumination (data not shown).

To evaluate the specificity of our mPCR for S. suis, field isolates of *Streptococcaceae*, *Pasteurellaceae*, and *Alcaligenaceae* commonly recovered from the upper respiratory tracts of pigs on farms in England and Wales were used as a panel of negative controls. No mPCR amplicons, after 35 thermal cycles and gel electrophoresis, were visible under UV transillumination for any of the panel of 10 negative controls (data not shown).

### Evaluation of our pathotyping tool with an out-of-sample collection.

**FIG 2:**
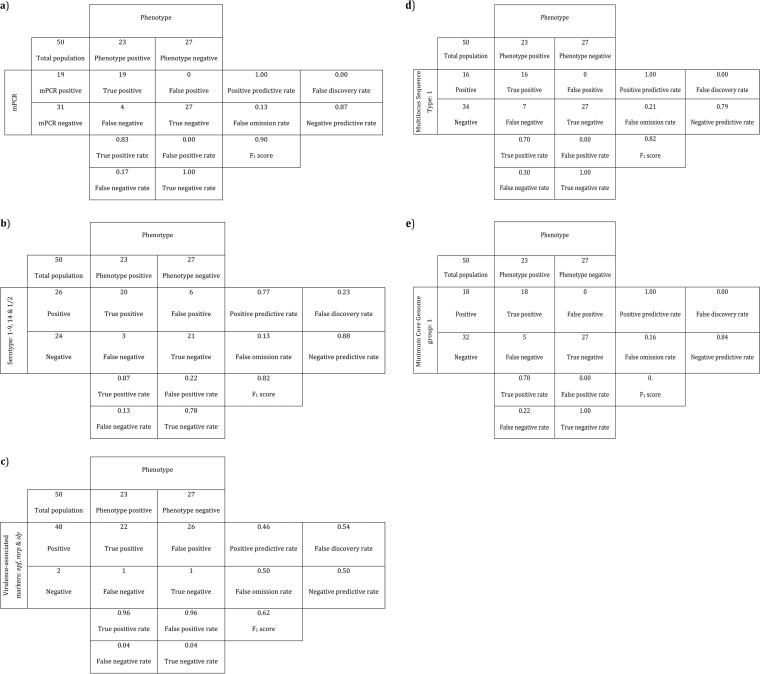
Contingency tables used to calculate the performance metrics summarizing the classification of Streptococcus suis isolates in the out-of-sample test collection (*n* = 50). Each panel compares the observed clinical phenotype, considered the gold standard in this study, to the use of (a) the newly described pathotyping markers, (b) serotypes (1 to 9, 14, and 1/2), (c) virulence-associated markers (epf, mrp, and/or sly), (d) multilocus sequence type (MLST) 1, and (e) assignment to minimum core genome (MCG) sequence type 1 as markers of invasive disease.

Further evaluation of our pathotyping tool (GLM and mPCR) was done using an out-of-sample test collection of 50 previously uncharacterized (genetically) S. suis isolates (23 invasive disease associated and 27 non-disease associated). Template DNA extracted from each of the 50 isolates produced the 722-bp mPCR amplicon corresponding to the S. suis species-specific marker SSU0577. For each isolate, the presence/absence of mPCR amplicons was then put into the GLM and the cutoff threshold of 0.43 applied to the fitted values to generate the binary classification decision. Figure 2a summarizes the classification of the out-of-sample test collection isolates in comparison to the observed clinical metadata, resulting in a sensitivity of 0.83, specificity of 1.00, negative predictive value of 0.87, and positive predictive value of 1.00.

### Comparison of our pathotyping tool to existing methods used to subtype disease-associated isolates of S. suis.

To compare our pathotyping tool to the use of serotype as a proxy to predict the virulence potential of S. suis isolates, the serotypes most frequently recovered from diseased pigs (1 to 9, 14, and 1/2) were considered a marker of disease association and all other serotypes considered markers of non-disease association. Figure 2b summarizes the classification of the out-of-sample test collection isolates in comparison to the observed clinical metadata and shows that the use of molecular serotypes 1 to 9, 14, and 1/2 to predict disease-association performed with a sensitivity of 0.87 (*n* = 3 type II errors), not statistically different from our new mPCR pathotyping tool (*P* value = 0.31731, McNemar’s chi-squared test for count data). Other performance metrics for the molecular serotype-based approach were a significantly worse positive predictive value, 0.77 (weighted generalized score statistic for comparison of predictive values, *P* value = 0.01149), and a significantly worse specificity, 0.78 (*n* = 8 type I errors; *P* value = 0.01431, McNemar’s chi-squared test for count data); no statistically significant difference in negative predictive value was observed (weighted generalized score statistic for comparison of predictive values, *P* value = 0.90553).

To compare our pathotyping tool to the use of *epf*, *mrp*, and/or *sly* for the identification of virulent S. suis strains, first a GLM was fitted to the prevalence data of these virulence-associated genes in the original training collection of S. suis isolates and, using the same selection criteria as previously described for the pathotyping markers, a ROC curve used to select the cutoff of 0.12 to convert the GLM fitted values into a binary class decision. The predict function, implemented in the R package *logistf* (47), was then used to generate fitted values for the isolates in the out-of-sample test collection (Table S1). Figure 2c summarizes the classification of the out-of-sample test collection isolates as invasive disease associated and non-disease associated based on the positive detection of *epf*, *mrp*, and/or *sly* in comparison to the observed clinical phenotype. The combined virulence-associated markers performed with a sensitivity of 0.96 (*n* = 1 type II errors), not statistically different from our new mPCR pathotyping tool (*P* value = 0.08326). Other performance metrics for the virulence-associated genotyping approach were a significantly worse positive predictive value, 0.46 (*P* value = 2.97708e^−7^; incidentally performing no better than chance [exact binomial test *P* value = 1]), and a significantly worse specificity, 0.04 (*P* value = 3.41417e^−7^). The negative predictive value was calculated to be 0.50, worse but not statistically significantly different (*P* value = 0.07853).

We compared our pathotyping tool to the use of the MLST scheme of King et al. (38) as a proxy to predict the virulence potential of S. suis isolates. Sequence type 1 (ST1) was assigned to 70% of disease-associated isolates and 3% of non-disease-associated isolates of the training collection (Table S1). As ST1 is mostly associated with disease in both pigs and humans in Europe (12), we used assignment to ST1 as a binary classifier to indicate disease association in comparison to the observed clinical metadata. Figure 2d summarizes the classification of the out-of-sample test collection isolates as invasive disease associated and non-disease associated based on the assignment to ST1 in comparison to the observed clinical phenotype. Assignment to ST1 performed with a sensitivity of 0.70 (*n* = 7 type II errors), worse than our pathotyping tool but not a statistically significant difference (*P* value = 0.08326). The negative predictive value was calculated to be 0.79, again worse but not a statistically significant difference (*P* value = 0.08294). Other performance metrics (specificity and positive predictive value) were found to be identical to our pathotyping tool.

Finally, we compared our pathotyping tool to the use of the MCG typing scheme of Zheng et al. (33, 37), one of the most recent typing schemes that exploits advances in next-generation sequencing to identify virulent S. suis strains. MCG group 1 was assigned to 77% of disease-associated isolates and 3% of non-disease-associated isolates of the training collection (Table S1). Together with the report of MCG group 1 being assigned to all highly virulent isolates tested during design of the typing scheme (33), we used assignment to MCG group 1 as a binary classifier to indicate disease association. Figure 2e summarizes the performance of MCG group 1 in comparison to the observed clinical metadata. Assignment to MCG group 1 performed with a sensitivity of 0.78 (*n* = 5 type II errors), again worse than our pathotyping tool but not a statistically significant difference (*P* value = 0.31731). The negative predictive value was calculated to be 0.84, also worse but not statistically significantly different (*P* value = 0.31725). Other performance metrics (specificity and positive predictive value) were found to be identical to our pathotyping tool.

## DISCUSSION

We have described the design of a pathotyping tool (GLM and mPCR) exploiting the identification of genetic markers in the S. suis accessory genome (i.e., genes absent from one or more isolates or unique to a given isolate) associated with the observed clinical phenotypes (i) invasive disease and (ii) asymptomatic carriage on the palatine tonsils of pigs on UK farms. Initial analyses of the original training collection were unable to identify any single genetic marker of invasive disease prevalent in >95% of invasive disease-associated isolates and not positively identifiable in <5% of non-disease-associated isolates. Furthermore, we found that over half (*n* = 40) of published putative virulence factors, extracted from the previous comprehensive review by Fittipaldi et al. (24) and present in P1/7, did not show a strong relationship with observed clinical phenotype, as they were either (i) positively detected in the S. suis core genome (i.e., prevalent in all isolates; *n* = 38) or (ii) not detected by our methods in any of the 115 isolates of the training collection (*n* = 2 [data not shown]). The reason for this is unclear, although it could be an effect of previous studies being limited to small numbers of isolates often restricted to serotype 2 (58) or of varied and inconsistent animal models between research groups (25).

To avoid restricting our analyses to previously published reports and not taking full advantage of the statistical power of our WGS data set, we used two complementary genome-wide association studies and then logistic regression analysis for the final selection of genetic markers to pathotype S. suis. Using logistic regression analysis also allowed for the possibility that multiple genetic markers might best describe the S. suis pathotype. Our pathotyping markers assigned the 115 S. suis isolates of the original training collection to phenotypic groups (disease associated and non-disease associated) with a sensitivity of 0.91 (i.e., the proportion of isolates recovered from systemic sites and predicted to be disease-causing isolates), a specificity of 0.79 (i.e., the proportion of isolates recovered from the upper respiratory tract of pigs without any typical signs of S. suis infection and predicted to be non-disease-associated isolates), a negative predictive value of 0.91 (i.e., the proportion of isolates predicted to be non-disease associated that were actually recovered from the upper respiratory tract of pigs without any typical signs of S. suis infection), and finally, a positive predictive value of 0.79 (i.e., the proportion of isolates predicted to be associated with invasive disease that were actually recovered from a systemic site).

An important caveat of our pathotyping tool design is consideration of the observed clinical phenotype associated with each isolate as the gold standard to characterize S. suis isolates as disease associated or non-disease associated. In the absence of an agreed-upon superior approach, clinical metadata were used to assign S. suis isolates to one of two phenotypic groups; it is acknowledged that such an approach is not perfect, as not all additional factors can be accounted for, such as host immune status, concurrent infections, or environmental conditions that could influence the susceptibility of a host to S. suis-associated disease. Indeed, reports of *in vivo* challenge studies can be readily found in the S. suis literature, although most describe data limited to a small number of isolates, often restricted to serotype 2 (58), and under very different conditions, making the extrapolation of findings difficult to interpret. An ideal standard would require an agreed-upon panel of isolates for which a series of consistently controlled experimental infection challenge studies had been undertaken using pigs of identical immune status and genetics. However, in order for this to happen, experts in the field must first agree on a suitable model and set of well-defined criteria to score virulence (25, 59, 60).

Another important caveat of our pathotyping tool design is the source of S. suis isolates of the original training collection that were deemed to be non-disease associated. While all efforts were made to accurately define invasive disease-associated and non-disease-associated phenotypic groups, it should be acknowledged that non-disease-associated isolates of the original training collection were recovered from routine submissions to the APHA in 2010 and that these pigs were not healthy, even though they did not show signs of typical streptococcal disease; instead, clinical features were consistent with different noninfectious diseases or disease caused by other, non-S. suis infectious agents. Indeed, 13 isolates of the original training collection deemed to be non-disease associated by phenotype were predicted by our pathotyping tool to have the potential to cause invasive disease. These 13 type I errors (or false positives) in comparison to the observed clinical metadata could in fact be true predictions and examples of S. suis strains with the potential to cause invasive disease being carried in the upper respiratory tract of pigs on UK farms. Therefore, it is possible that the mortality of these 13 pigs was due to clones of isolates recovered from the palatine tonsils or trachea-bronchus yet was not identified as so due to a concurrent or opportunistic infection presenting a more obvious phenotype, such as diarrhea. Such an observation is supported by evidence in the literature reporting that virulent strains of S. suis can be isolated from the tonsils of pigs without obvious streptococcal disease (61, 62), which is likely to represent carriage of invasive disease-causing strains by pigs that have mounted an effective immune response.

We deemed the false-negative rate (1 − sensitivity) to be the most valuable performance metric for a S. suis pathotyping tool in order to establish and maintain a pig population free of invasive disease-associated S. suis strains. During out-of-sample testing, the false-negative rate of 0.17 corresponded to four false negatives (or type II errors) where non-disease-associated pathotyping tool predictions were made for isolates linked with invasive disease clinical metadata. It is interesting to speculate at the reasons for such observations. Often S. suis strains are described as opportunistic or secondary pathogens that without a weakened host immune status (due to stress or concurrent infection) would normally be carried asymptomatically, contributing to the normal oral microflora of pigs. This may be the explanation for the differences observed between our pathotyping tool prediction and the observed clinical phenotype, again emphasizing the fallibility of the phenotype assigned when it is based on field sampling without carefully controlled infection challenge data.

Comparison to published methods revealed our molecular pathotyping tool to be a competitive method to subtype S. suis isolates, even though the necessarily small number of clinically phenotyped isolates in the out-of-sample collection limited the statistical power of the comparison. Comparing the commonly used performance metrics sensitivity, specificity, negative predictive value, and positive predictive value, we found the use of (i) serotypes 1 to 9, 14, and 1/2, (ii) a GLM based on the positive detection of virulence-associated markers *epf*, *mrp*, and/or *sly*, (iii) assignment to MLST 1, and (iv) assignment to MCG group 1 showed sensitivities statistically similar to that of our pathotyping tool. However, the trade-off for high sensitivities was significantly worse specificities and negative predictive values when using serotypes 1 to 9, 14, and 1/2 or the virulence-associated markers *epf*, *mrp*, and/or *sly*, in certain cases performing no better than chance (*P* value = 1) in comparison to our pathotyping tool. Overall, the performance of our pathotyping tool was at least statistically similar to and competitive with, and in some cases better than, previously described methods for assessing the clinical significance of S. suis isolates. Similarly, performance of our newly proposed S. suis species-specific marker (SSU0577) was encouraging. An important consideration regarding our pathotyping tool, due to the presence of S. suis-like organisms such as Streptococcus orisratti in the pig upper respiratory tract, we acknowledge that the specificity of SSU0577 for S. suis and not S. suis-like organisms needs to be extended and studied further against markers such as *recN*.

At present, the role in pathogenesis of our newly defined pathotyping markers is unknown. Based on predicted biological functions (Table 1), we speculate that marker SSU0207, predicted to be a copper-exporting ATPase, might allow S. suis to avoid copper toxicity inside phagocytes, as copper homeostasis has been shown to be important in many bacterial species (63–65). The marker SSU1589 is annotated as a type I restriction-modification (RM) system S protein in S. suis strain P1/7. Ubiquitous among prokaryotes, type I RM systems are large multifunctional protein complexes thought to defend host bacteria from foreign DNA borne by bacteriophages, and they have recently been described for P1/7 and S. suis strains isolated in the Netherlands (66, 67). Considered primitive immune systems in bacteria, it has been proposed that the range of functions RM systems may have should be expanded to include stabilizing mobile genetic elements or gene regulation, potentially providing evolutionary fitness advantages and virulence under certain conditions (68). Indeed, the proposed role in protection against foreign DNA may merely be a coincidental benefit of these functions (69). In fact, a type I RM system in Streptococcus pneumoniae which can undergo genetic recombination with truncated variants of the same gene to generate alternative variants with different methylation specificities could control global changes in gene expression (70). In Streptococcus pneumoniae there is a selection for variants of this genetic switching *in vivo*, indicating a role in systemic disease.

Our third genetic marker (SSUST30534), a putative sugar ABC transporter, was positively associated with the non-disease-associated phenotype (asymptomatic commensal-like carriage). The practical application of the genetic marker positively associated with asymptomatic carriage might not be immediately obvious, but its statistical significance in the GLM is noteworthy. Indeed, gene loss (of so-called antivirulence genes) in the evolution of bacterial pathogens from nonpathogenic commensal strains could be a mechanism of fine-tuning pathogen genomes for maximal fitness in new host environments; in short, when regulation of invasion, replication, and transmission processes is altered, virulence can emerge (71). Indeed, genome reduction via gene loss and pseudogenization associated with enhanced pathogenicity has been described for other bacteria, such as *Rickettsia* spp., *Shigella* spp., and *Yersinia* spp. (71). Genome reduction through the loss of genes, potentially interfering with host infection, has also been proposed for S. suis (35). Therefore, as the elimination of the genetic marker associated with asymptomatic carriage from the GLM could not be done without a statistically significant loss of fit, it was retained and its usefulness was evaluated.

In conclusion, we foresee a useful clinical application of our pathotyping tool in preventative programs aimed at monitoring the health status of pigs and identification of subclinical carriers of invasive disease-associated S. suis strains in the upper respiratory tract. Our approach can easily be updated to incorporate global strain collections (such as strains from North America and Southeast Asia) to identify geographically dependent phenotypes. This could contribute to a lower prevalence of disease attributed to S. suis among pig populations and consequently a reduction in the usage of antibiotics in the swine industry, as well as a reduction in zoonotic transmission of this pathogen through improved surveillance of pig populations.

## Supplementary Material

Supplemental file 1

Supplemental file 2

Supplemental file 3
